# Impact of SARS-CoV-2 on Severe Asthma Patients Undergoing Biological Therapy: A Multicenter Study

**DOI:** 10.3390/jcm14217583

**Published:** 2025-10-25

**Authors:** Marina Lampalo, Branislava Milenkovic, Anamarija Stajduhar, Iva Lucija Burnac, Vesna Tomic Spiric, Ivana Stankovic, Zorica Lazic, Bojan Djokic, Dragan Vukosavljevic, Sanja Dimic Janjic, Aleksandra Plavsic, Borislav Bozanic, Eugenija Kasap Basioli, Bojan Miletić, Sanja Hromiš, Hana Safic Stanic

**Affiliations:** 1 University Hospital Center Zagreb, 10000 Zagreb, Croatia; anamarija.stajduhar@kbc-zagreb.hr; 2 Faculty of Health Studies, University of Rijeka, 51000 Rijeka, Croatia; bojan.miletic@uniri.hr; 3 Clinic for Pulmonary Diseases, University Clinical Centre of Serbia, 11000 Belgrade, Serbia; branislava.milenkovic@kcs.as.rs (B.M.); drvukosavljevic993@live.com (D.V.); plucnebolestikcs@kcs.ac.rs (S.D.J.); 4 Faculty of Medicine, University of Belgrade, 11000 Belgrade, Serbia; vesnatomicspiric63@gmail.com (V.T.S.); aleksandra.plavsic@gmail.com (A.P.); 5 Clinical Hospital Sveti Duh, 10000 Zagreb, Croatia; ivalucija.burnac@gmail.com; 6 Clinic for Allergy and Immunology, University Clinical Centre of Serbia, 11000 Belgrade, Serbia; 7 Clinic for Lung Diseases, University Clinical Centre of Nis, 18000 Nis, Serbia; staivana@gmail.com (I.S.); borislavbozanic@gmail.com (B.B.); 8 Faculty of Medicine, University of Nis, 18000 Nis, Serbia; 9 Clinic for Pulmonology, University Clinical Centre Kragujevac, 34000 Kragujevac, Serbia; zoricalazickg@gmail.com (Z.L.); bojan.djokic@gmail.com (B.D.); 10 Faculty of Medicine, University of Kragujevac, 34000 Kragujevac, Serbia; 11 General Hospital Zadar, 23000 Zadar, Croatia; ebasiolikasap@unizd.hr; 12 Institute for Pulmonary Diseases of Vojvodina, 22000 Sremska Kamenica, Serbia; sanja.hromis@institut.rs; 13 Faculty of Medicine, University of Novi Sad, 21000 Novi Sad, Serbia; 14 Croatian Institute of Transfusion Medicine, 10000 Zagreb, Croatia; hsafic@gmail.com

**Keywords:** severe asthma, COVID-19 outcomes, biological therapy, vaccination, respiratory infection

## Abstract

**Background**: The SARS-CoV-2 pandemic raised concerns about severe asthma (SA) patients, especially those on biological therapy. While initial fears of increased risks diminished for the general asthma population, a subset of severe cases undergoing specific treatments continued to be closely monitored. Our study aimed to evaluate COVID-19 occurrence, asthma exacerbations, hospitalizations, and outcomes in severe asthma patients. **Methods**: This multicenter study gathered data from 237 adult severe asthma patients in Croatia and Serbia to assess COVID-19 impact compared to the general population. Participants received omalizumab, mepolizumab, benralizumab, or reslizumab. Data on demographics, clinical features, and COVID-19 were collected. COVID-19 symptoms and diagnostic testing were assessed as described. **Results**: No demographic differences were seen between COVID-19-positive and -negative groups, with eosinophilic asthma prevailing. Among patients with SA treated with biologics, the occurrence of COVID-19 did not differ significantly from that in the general population, and biologic therapy was not associated with more severe disease or higher mortality. Importantly, no meaningful differences were observed among different biologics regarding COVID-19 outcomes. Vaccination was associated with a reduction in severe cases and hospitalizations. **Conclusions**: Biologic therapy for severe asthma appears safe during the COVID-19 pandemic. Patients receiving biologics did not experience worse outcomes than the general population, and no biologic was linked to poorer COVID-19 prognosis. Vaccination further contributed to protection against severe disease.

## 1. Introduction

With the start of the SARS-CoV-2 pandemic, many new concerns have arisen, among others, of whether severe asthma (SA) patients, especially those treated with biological therapy, are at a greater risk of infection and complications. Earlier studies found that the outcomes of COVID-19 varied greatly, from asymptomatic to lethal, and biological treatments of some other inflammatory diseases did not confer a greater risk of COVID-19 complications [1,2]. Elderly patients, obese, diabetics, and cardiovascular patients, on the other hand, were especially endangered [1]. Fortunately, studies that have been published so far have demonstrated that asthma patients do not have a greater prospect of severe COVID-19 compared to the general population, and SARS-CoV-2 infection does not automatically mean that there is asthma exacerbation, potentially due to treatment with inhalation corticosteroids or better hygiene in this population [1,3,4]. However, papers concerning SA patients have shown that this subgroup is at a greater risk of adverse events, perhaps due to oral corticosteroid therapy, certain phenotype, uncontrolled asthma, or biological asthma therapy which impedes the protective type 2 inflammatory response to SARS-CoV-2 [1,5,6,7]. The invention of vaccines against COVID-19 offered a tool against the severe form of infection, but some questions remain to be answered. For instance, we still have no definite knowledge of vaccine–biologics interactions, the number of boosters needed, the frequency of vaccination, and the short- and long-term effects on SA control, although some recent study results show that mRNA SARS-CoV-2 vaccines in SA patients are safe and have no negative effects on quality of life in this asthma population [8]. On the other hand, poor asthma control was a contraindication for SARS-CoV-2 vaccination in most studies, and there is currently no definite stance on non-mRNA vs. mRNA SARS-CoV-2 vaccine recommendations.

The aim of this multicenter, observational, cross-sectional study was to evaluate the occurrence and clinical course of COVID-19 in patients with severe asthma treated with biologics. Specifically, we assessed the frequency of asthma exacerbations during SARS-CoV-2 infection, the proportion of patients requiring hospitalization, oxygen supplementation, or ventilatory support, and the incidence of severe COVID-19 and COVID-19-related death. Where possible, these outcomes were interpreted in the context of epidemiological data from the general populations of Croatia and Serbia.

## 2. Methods

### 2.1. Participants and Study Design

Participants over the age of 18 years with the diagnosis of SA according to the ERS/ATS guidelines were included in this study [9]. COVID-19 cases were identified through a cross-sectional survey between 2020 and 2022 among adults with SA. A total of 237 patient agreed to take part in this investigation, and signed the informed consent: 143 from the Clinic for Pulmonary Diseases, University Hospital Center Zagreb, 25 from the Clinic for Pulmonary Diseases, University Center of Serbia, 25 from the Clinic for Pulmonary Diseases, Clinical Center of Kragujevac, 18 from the Clinic of Allergy and Immunology, University Center of Serbia, 16 from the Clinic for Pulmonary Diseases, University Hospital Center Rijeka, and 10 from the Clinic for Pulmonary Diseases, University Clinical Center Nis.

The local clinical research ethics committees in participating hospitals approved the project under the numbers (Croatia: class 8.1-19/300-2, No. 02/21 AG; Serbia: No. 915/3). The rights, safety, and well-being of clinical investigation subjects were protected consistently with the ethical principles that have their origin in the Declaration of Helsinki.

All participants were treated with biological therapy for SA. The monoclonal antibodies available for clinical use in SA in these countries are as follows: omalizumab, mepolizumab, benralizumab, and reslizumab. None of the patients discontinued biological therapy during COVID-19 infection.

Demographic data, clinical characteristics, asthma phenotype, laboratory and radiological findings, pulmonary function test, chronic therapy, and changes in clinical control by the Asthma Control Test (ACT), were obtained by reviewing the electronic medical record system. Allergic asthma was characterized by early onset, elevated IgE, and positive allergen tests; eosinophilic asthma was characterized by adult onset, elevated blood/sputum eosinophils, and no atopy; while mixed phenotype shows features of both with elevated IgE and eosinophils. A COVID-19 diagnosis was defined as a positive PCR test for SARS-CoV-2, positive SARS-CoV-2 antigen rapid diagnostic tests (Ag-RDTs), typical symptoms <10 days after contact with a confirmed case, or typical symptoms with positive SARS-CoV-2 serology results afterwards in case the PCR test was not available at the time of initial diagnosis. Recovery from COVID-19 was defined as resolution of fever for at least 48 h without antipyretics, improvement in respiratory symptoms, and completion of isolation in accordance with national guidelines.

A standardized questionnaire was administered by phone or during outpatient visits and data about COVID-19 (vaccination status, symptoms, treatment-drugs, oxygen therapy, mechanical ventilation, course of disease and outcome, diagnostic testing, and exacerbations) were obtained. Vaccination status was categorized according to the number of doses received (one dose, two doses, or three or more, i.e., booster).

Treatment for asthma included the following: monoclonal antibodies (omalizumab, mepolizumab, reslizumab, benralizumab, and dupilumab), inhaled corticosteroids (ICS; budesonide, fluticasone, and mometasone), long-acting β2-agonists (LABA; formoterol and salmeterol), long-acting muscarinic antagonists (LAMA; tiotropium), anti-leukotrienes (montelukast), systemic corticosteroids (prednisone, methylprednisolone; dose recorded), and other drugs (for example azithromycin). Therapy duration was calculated from treatment initiation until either a COVID-19 diagnosis (for COVID-19-positive patients) or until the last follow-up during the pandemic period (for COVID-19-negative patients).

Symptoms suggestive of COVID-19 were as follows: dyspnoea, rhinorrhea, productive cough, headache, chest pain, fever, sudden olfactory and gustative dysfunction, malaise, headache, muscle pain, and joint pain.

Diagnostic testing for COVID-19 included nasopharyngeal swab (PCR), SARS-CoV-2 Ag-RDTs, serology (SARS-CoV-2 IgG), chest x-ray, and chest CT scan. IgG serology was used only in cases where PCR testing was not available at the time of initial diagnosis, and vaccination status was carefully recorded to minimize potential misclassification bias. Post-vaccination IgG positivity was not considered diagnostic of COVID-19 infection.

### 2.2. Statistical Analysis

Statistical analysis was conducted using descriptive and inferential methods. For distribution estimation, the Kolmogorov–Smirnov test was employed, and parametric or non-parametric statistical tests were chosen accordingly. Data were presented as means ± standard deviations for normally distributed variables and as medians (25th–75th percentile values) for non-normally distributed variables. Categorical data were compared using the Chi-square test with the Fisher exact test where appropriate, and for continuous variables with multiple groups, the parametric ANOVA test with Tukey test as a post hoc test or the Kruskal–Wallis ANOVA with the Mann–Whitney U test were utilized for three-group comparisons. For two group comparisons, the Mann–Whitney U test was employed. A *p* value less than 0.05 was considered statistically significant for all tests. The statistical program used was IBM SPSS ver. 18.0 (IBM, Armonk, New York, NY, USA).

## 3. Results

The general and clinical characteristics of the study subjects are presented in Table 1, highlighting differences between the COVID-19-negative and -positive groups.

The distribution of patients across different institutions differed significantly between the COVID-19-negative and -positive groups (χ^2^ = 29.1, *p* < 0.001). The Pulmology clinic in Zagreb had the highest number of patients in both groups. The mean age of the patients was 55.3 ± 13.2, with no significant difference between groups (56.7 ± 13.6 vs. 53.7 ± 12.6 years, *p* = 0.088); however, patients with COVID-19 were slightly younger than patients without the disease. Regarding gender, 85 patients (35.9%) were male and 152 patients (65.1%) were female. The gender distribution did not show a statistically significant difference between groups (*p* = 0.683); however, the majority of patients in both groups were female. The distribution of asthma phenotypes (allergic, eosinophilic, and combined) did not show a statistically significant difference between the COVID-19-negative and -positive groups (*p* = 0.212), although the eosinophilic phenotype prevailed. The use of different biological therapies (benralizumab, mepolizumab, omalizumab, and reslizumab) was comparable between the two groups (χ^2^ = 0.824, *p* = 0.844). Benralizumab was the most commonly used biological therapy overall. The mean duration of therapy was 21.8 ± 15.8 months with no significant difference between the COVID-19-negative and -positive groups (21.6 ± 15.7 vs. 22 ± 16 months, *p* = 0.847).

The frequency of asthma exacerbations did not significantly differ between the two groups (χ^2^ = 5.5, *p* = 0.159). The majority of patients experienced 0–2 exacerbations. Regarding asthma exacerbation during COVID-19 disease, 74 patients had no exacerbation, 30 patients had one exacerbation, and 1 patient had two exacerbations during the disease. The majority of the patients had no or just one asthma exacerbation after recovering from COVID-19.

There was a borderline significant trend towards a higher proportion of patients receiving oral corticosteroid therapy in the COVID-19 group compared to the negative group—35 vs. 30 patients (χ^2^ = 3.2, *p* = 0.051). The mean predicted FEV1 was 71.7 ± 22.5 with no significant difference between the groups (71.7 ± 22.5 vs. 71.1 ± 23.6, *p* = 0.728). The distribution of comorbidities, including chronic rhinitis, chronic sinusitis, nasal polyps, hypertension, diabetes mellitus, other pulmonary diseases, and other extra-pulmonary diseases, did not significantly differ between the two groups (χ^2^ = 5.5, *p* = 0.479). Table 1 lists various COVID-19-related symptoms experienced by asthma patients. Most patients experienced dyspnea (40.0%), rhinorrhea (40.0%), and dry cough (47.6%). Other symptoms named in Table 1 were also present. The mean duration of COVID-19-related symptoms experienced by asthma patients was 8.9 ± 6.7 days. Only 15% of COVID patients were hospitalized, and the hospitalization duration was predominantly up to 2 weeks. Biological therapy duration was the longest for the mepolizumab group of patients (median: 30 months) and the shortest for the benralizumab group (median: 13 months). Mepolizumab and reslizumab groups of patients had a significantly lower number of exacerbations prior to COVID-19. Patients devoted to the use of benralizumab therapy had a significantly higher number of eosinophilic forms of asthma, while patients from the omalizumab group predominantly had an allergic type of the disease (Table 2).

A patients’ comparison according to COVID-19 severity indicated significantly lower FEV1 in patients with more severe disease. These patients also had a higher percentage of oxygen, systemic corticosteroids, or both therapies compared to patients with mild forms of the disease, as in the case of LMWH (low-molecular-weight heparin) therapy (Table 3). There was no difference in vaccinal status, number of hospitalized subjects, and therapy type in different quarters’ periods of the pandemic (Table 4). Chi-square analysis showed a significantly higher number of COVID-19 patients before vaccination in the earlier period of the pandemic, but the opposite was true for the later pandemic periods, when more patients were vaccinated (Table 5).

## 4. Discussion

The COVID-19 pandemic has had a significant impact on public health worldwide and individuals with underlying respiratory conditions have been identified as a vulnerable population. At the start of the pandemic, there was concern that SARS-CoV-2 may act as a trigger for asthma exacerbations, in a similar manner to other respiratory viruses. Some studies have reported that pre-existing asthma, particularly SA, could be associated with an increased risk of experiencing more severe illness and adverse events if they acquire COVID-19, a higher risk of hospitalization, intensive care unit (ICU) admission, and mortality due to COVID-19 [10]. The underlying chronic airway inflammation and potential impaired lung function in SA patients might contribute to this heightened risk. Furthermore, COVID-19 may act as a trigger for asthma exacerbations in individuals with SA, by increased airway inflammation and bronchial hyperresponsiveness, potentially exacerbating asthma symptoms [11].

On the other hand, recent studies have shed different light on the impact of COVID-19, specifically on SA patients who are undergoing various biologics. Surprisingly, these studies have shown that the presence of COVID-19 in such individuals does not necessarily lead to an increased risk of hospitalization, admission to the intensive care unit (ICU), or death. In other words, SA patients undergoing different biologics do not appear to have a higher susceptibility to severe COVID-19 outcomes or greater disease severity and mortality compared to the general population. The study by Numata et al. supports this finding, suggesting that biologic therapies for severe asthma do not adversely affect the clinical course of COVID-19. There were no differences among biological drugs used as well [12,13,14,15].

We observed no significant demographic or gender differences between the COVID-19-negative and -positive groups. However, the COVID-19-positive patients were slightly younger on average, and both groups predominantly consisted of female respondents, aligning with findings from a comparable study [14]. The average age of COVID-19-positive SA patients aligns with the general population’s median age, which ranged from 40 to 50 years during the pandemic while the general population exhibited a more balanced gender distribution or a slight female predominance [16,17].

The distribution of asthma phenotypes remained consistent in SA patients with and without COVID-19 infection suggesting that the presence of COVID-19 does not appear to alter the distribution of asthma phenotypes in SA patients. The eosinophilic phenotype appears to prevail in both groups, emphasizing the importance of targeted therapies for eosinophilic-driven inflammation.

The use of different biologics was comparable between the COVID-19-negative and -positive groups, with benralizumab being the most commonly used overall. This finding aligns with some previous studies. Xie et al. found that the use of biological therapies (such as omalizumab and mepolizumab) did not significantly differ between COVID-19-positive and -negative asthma patients [18]. This suggests that the choice of biological therapy may not have a significant impact on COVID-19 susceptibility or severity. The current study reported no significant difference in biologic therapy duration between the groups, as well. Similarly, a study by Jackson et al. observed no difference as well, suggesting that the duration of biological therapy may not be influenced by COVID-19 status [19] but depends on various factors, such as patient characteristics, disease severity, and treatment response. The efficacy of individual biologics in reducing exacerbations and improving asthma control is well established [20,21]. Our study confirmed expected patterns of biologic use in relation to asthma phenotype, with benralizumab predominantly prescribed to patients with eosinophilic asthma and omalizumab to those with allergic asthma. These associations reflect established prescribing indications rather than new findings of this study. Importantly, when comparing outcomes during COVID-19, no significant differences were observed between biologics, supporting the conclusion that all currently approved biologics appear similarly safe in the context of SARS-CoV-2 infection [22,23].

The frequency of asthma exacerbations was similar between the COVID-19-positive and -negative groups, with the majority of patients experiencing 0–2 exacerbations (χ^2^ = 5.5, *p* = 0.159). The number of asthma exacerbations also remained constant during and after COVID-19 infection. Published data confirm this finding which implies that the COVID-19 infection itself may not directly increase the risk of asthma exacerbations [1,3,4,24]. There was a statistically insignificant higher proportion of patients receiving oral corticosteroid therapy in the COVID-19-positive group compared to the control group (χ^2^ = 3.2. *p* = 0.051). Fung et al. also reported a higher prevalence of oral corticosteroid use in COVID-19-positive asthma patients, indicating that COVID-19 infection may increase the need for systemic corticosteroid treatment in some individuals with asthma [25]. No significant difference was observed in the percentage of predicted FEV1 between the groups or in the distribution of comorbidities, including chronic rhinitis, chronic sinusitis, nasal polyps, hypertension, diabetes mellitus, other pulmonary diseases, and other extra-pulmonary diseases. A systematic review and meta-analysis by Halpin et al. indicated that COVID-19 infection did not significantly affect lung function in individuals with pre-existing respiratory conditions, including asthma [26]. This implies that COVID-19 has a limited impact on lung function changes among asthma patients, and asthma itself, rather than specific comorbidities, which may pose a significant risk factor for COVID-19 infection [27].

Dyspnea, rhinorrhea, and dry cough were identified as common symptoms in SA patients with COVID-19, along with other symptoms such as headache, weakness, chest pain, fever, gastrointestinal disturbances, and back pain. A study by Jackson et al. conducted a systematic review and meta-analysis and found that dyspnea was one of the most common symptoms reported [19]. Rhinorrhea and dry cough were also frequently reported symptoms, with prevalences of 7% to 43% and 11% to 61%, respectively. Furthermore, the presence of additional symptoms such as headache, weakness, chest pain, fever, gastrointestinal disturbances, and back pain among SA patients with COVID-19 highlights the systemic nature of the disease, unrelated to asthma itself [28]. These findings suggest that COVID-19 can affect multiple organ systems and lead to a diverse array of symptoms in SA patients. The mean duration of COVID-19-related symptoms experienced by asthma patients was 8.9 ± 6.7 days, which falls within the range observed in the general population [29,30]. Studies have reported variable durations of symptoms in COVID-19 patients, reflecting the heterogeneity of the disease, and the influence of various factors, including the severity of the disease, the individual’s immune response, and the presence of comorbidities.

Only 15% of COVID patients were hospitalized, and the hospitalization duration was predominantly from 1 to 2 weeks, and only 2 patients out of 105 required mechanical ventilation for between 7 and 15 days, respectively. A study by Zhou et al. conducted among COVID-19 patients with comorbidities such as asthma reported a 14% hospitalization rate [31]. Another study by Wu et al. found that asthma was not significantly associated with an increased risk of severe COVID-19 outcomes [32]. These findings again support the notion that asthma alone does not necessarily confer a higher risk of severe disease or hospitalization in COVID-19 patients. SA patients receiving biological therapies did not experience increased COVID-19 severity, hospitalizations, or mortality compared to the general population [33,34].

The study also found that patients with more severe COVID-19 disease had a higher usage of oxygen therapy and systemic corticosteroids. These findings are consistent with current treatment guidelines for severe COVID-19 cases, which recommend the use of oxygen therapy and systemic corticosteroids to manage respiratory distress and reduce inflammation [35,36]. LMWH is commonly used as a prophylactic treatment to prevent blood clot formation in hospitalized patients with severe COVID-19 [24,37]. Ambulatory-treated patients did not require the use of LMWH, while LMWH is administered to hospitalized patients (10, 66.6%) according to protocol. However, contraindications for the use of LMWH were identified in five (33.3%) hospitalized patients, and it was therefore not administered.

Interestingly, we did not find any differences in the vaccination status, the number of hospitalized subjects, or therapy type across different quarters of the pandemic. This suggests that vaccination efforts and therapeutic interventions remained consistent throughout the different phases of the pandemic, indicating a standardized approach to COVID-19 management in asthma patients. Chi-square analysis showed a significantly higher number of COVID-19 patients before vaccination in the earlier period of the pandemic, but the opposite was true for the later pandemic periods, when more patients were vaccinated.

In summary, the study found no significant differences in age, gender, asthma phenotypes, or therapy duration between the COVID-19-negative and -positive groups. The frequency of asthma exacerbations did not significantly differ between the two groups. COVID-19-related symptoms were experienced by asthma patients for a mean duration of 8.9 days. The severity of COVID-19 was associated with decreased lung function, increased use of oxygen, and systemic corticosteroid therapy.

## 5. Conclusions

In conclusion, patients with severe asthma treated with biologics did not experience worse COVID-19 outcomes compared to the general population. Different biologic agents were associated with comparable safety profiles and similar clinical outcomes during SARS-CoV-2 infection. These findings support the continued use of biologics in the management of severe asthma throughout the COVID-19 pandemic. Further research is warranted to better understand the long-term effectiveness, safety, and optimal duration of biological therapies in asthma patients, particularly in the context of COVID-19.

Limitations: The cross-sectional design prevents inference of causality and does not allow for long-term outcomes to be assessed. Only patients with severe asthma were included, so results cannot be generalized to non-severe asthma patients.

## Figures and Tables

**Table 1:** General and clinical characteristics of study subjects, COVID-19-related data.

Variable	Whole Group of Patients	COVID-19 Negative N = 132	COVID-19 Positive N = 105	*p*
Institution				χ^2^ = 29.1 *p* < 0.001
Belgrade, Pulmonology clinic	25 (10.5)	7 (28.0)	18 (72.0)	
Belgrade, Alergology clinic	18 (7.6)	15 (83.3)	3 (16.7)	
Kragujevac	43 (18.1)	23 (53.5)	20 (46.5)	
Niš	10 (4.2)	8 (80.0)	2 (20.0)	
Rijeka	15 (6.8)	15 (100)	0 (0)	
Zagreb	125 (52.7)	63 (50.4)	62 (49.6)	
Age, years ^&^	55.3 ± 13.2	56.7 ± 13.6	53.7 ± 12.6	0.088
Gender				χ^2^ = 0.246 *p* = 0.683
Male	85 (35.9)	49 (57.6)	36 (42.4)	
Female	152 (65.1)	83 (54.6)	69 (45.4)	
Asthma phenotype				χ^2^ = 3.1 *p* = 0.212
Allergic	49 (20.7)	23 (17.6)	26 (24.8)	
Eosinophilic	107 (45.1)	65 (49.6)	41 (39.0)	
Combined	81 (34.2)	43 (32.8)	38 (36.2)	
Biological therapy				χ^2^ = 0.824 *p* = 0.844
Benralizumab	105 (44.3)	61 (58.1)	44 (41.9)	
Mepolizumab	45 (19.0)	25 (57.8)	19 (42.2)	
Omalizumab	66 (27.8)	34 (51.5)	32 (48.5)	
Reslizumab	21 (8.9)	11 (52.4)	10 (47.6)	
Therapy duration (months) ^&^		21.6 ± 15.7	22.0 ± 16.0	0.847
mean ± SD,	21.8 ± 15.8			
min–max,	(0–78)			
median (25th–75th percentile)	18 (9–32)			
Asthma exacerbation before COVID infection	0–12			χ^2^ = 5.5 *p* = 0.159
	0	67 (51.1)	55 (52.4)	
	1	28 (21.4)	32 (30.5)	
	2–5	31 (23.7)	14 (13.3)	
	>6	5 (3.8)	4 (3.8)	
Oral corticosteroid therapy				χ^2^ = 3.2 *p* = 0.051
No	172 (72.6)	102 (77.1)	70 (66.7)	
Yes	65 (27.4)	30 (22.9)	35 (33.3)	
FEV1 (% predicted), mean ± SD (min–max) ^&^	71.7 ± 22.5 (20–136)	71.1 ± 23.6	72.1 ± 21.2	0.728
Comorbidity				χ^2^ = 5.5 *p* = 0.479
Chronic rhinitis	49 (20.8)	30 (22.9)	19 (18.1)	
Chronic sinusitis	66 (28.0)	49 (37.4)	17 (16.2)	
Nasal polyps	58 (24.6)	39 (29.8)	19 (18.1)	
HTA	91 (38.6)	68 (51.9)	23 (21.9)	
Diabetes Mellitus	26 (11.0)	17 (13.0)	9 (8.6)	
Other pulmonary diseases	27 (11.4)	21 (16.0)	6 (5.7)	
Other extra-pulmonary diseases	124 (52.5)	93 (71.0)	31 (29.5)	
PCR confirmed diseases	/	/		/
No			34	
Yes			71	
Antigen test confirmed	/	/		/
No			67	
Yes			38	
Serological confirmation	/	/		/
No			103	
Yes			2	
Time of COVID-19 disease (by quartals)	/	/		/
1			4 (1.7)	
2			32 (13.5)	
3			14 (5.9)	
4			55 (23.2)	
Asthma exacerbation during COVID disease (number of times)	/	/		/
0			74	
1			30	
2			1	
COVID-19 related symptoms	/	/		/
Dyspnoea			42 (40.0)	
Rhinorrhoea			42 (40.0)	
Dry cough			50 (47.6)	
Productive (wet) cough			26 (24.8)	
Headache			41 (39.0)	
Weakness			61 (58.1)	
Chest pain			29 (27.6)	
Fever			78 (74.3)	
Gastrointestinal disturbances			25 (23.8)	
Back pain			30 (28.6)	
Other #			7 (6.7)	
COVID-19 related symptoms duration	/	/		/
mean ± SD,			8.9 ± 6.7	
min–max,			0–30	
median (25th–75th percentile)			7 (4–10)	
Asthma exacerbation after COVID infection	/	/		/
No			74 (70.5)	
once			30 (28.5)	
twice			1 (1.0)	
Oxygen and/or corticosteroid therapy	/	/		/
No			81 (77.1)	
O_2_			1 (1.0)	
Corticosteroid drug			20 (19.0)	
O_2_ + corticosteroid drug			3 (2.9)	
Oxygen therapy duration (0–25 days)	/	/		/
No			96 (91.4)	
<7 days			4 (3.8)	
<14 days			3 (2.9)	
>15 days			3 (2.9)	
Systemic corticosteroids	/	/		/
No			68 (64.8)	
Yes			37 (35.2)	
Systemic corticosteroids therapy duration (0–45 days)				
No			68 (64.8)	
<7 days			7 (6.7)	
<14 days			8 (7.6)	
>21 days			2 (1.9)	
>22 days			4 (3.8)	
Missing data			16 (15.2)	
LMWH (low-molecular-weight heparin)				
No			94 (89.5)	
Yes			11 (10.5)	
Other therapy (cipla inhalation, insulin)				
No			102 (97.1)	
Yes			3 (2.9)	
Quarters of COVID-19, n (%)		/		/
	First		4 (3.8)	
	Second		32 (30.5)	
	Third		14 (13.3)	
	Forth		55 (52.4)	
Hospitalization during COVID-19	32 (30.5)	/		/
No			90 (85.0)	
Yes			15 (15.0)	
Hospitalization duration (1–45 days)	14 (13.3)			
<7 days			5	
8–14 days			5	
15–21 days			3	
>22 days			2	
Noninvasive ventilation/mechanical ventilation	55 (52.4)			
No			105/103	
Yes			0/2 *	
Vaccine status				χ^2^ = 0.463 *p* = 0.547
No	59	35 (26.5)	24 (22.8)	
Yes	178	96 (73.5)	81 (77.2)	
Vaccine according to manufacturer				χ^2^ = 0.4.1 *p* = 0.660
AstraZeneca	23 (12.9)	11	12	
Moderna	17 (9.6)	9	8	
Pfizer	84 (47.2)	52	32	
Sinopharm	38 (21.3)	17	21	
Sputnik	11 (6.2)	6	5	
Johnson	3 (1.7)	1	2	

^&^ Mann–Whitney U test for continuous variables; χ^2^ test for categorical variables; # Myalgia, anosmia, ageusia, and sweating; * mechanical ventilation duration 7 and 15 days, respectively. Quarter periods: I to September 2020; II October 2020–March 2021; III April 2021–October 2021; and IV November 2021–April 2022.

**Table 2:** Influence of biological therapy type on clinical features and COVID-19-related conditions.

Parameter	Benralizumab	Mepolizumab	Omalizumab	Reslizumab	*p*
Age, years	55.1 ± 12.6	55.5 ± 13.2	54.6 ± 14.2	58.6 ± 13.6	0.676
FEV1%	75.2 ± 23.6	66.8 ± 22.6	70.0 ± 21.8	69.4 ± 16.4	0.155
Therapy duration (months)	13 (7–24)	30 (20–33) ^aaa^	20 (9–33) ^aa,b^	27 (7–44) ^a^	0.002
Exacerbation number	0–12	0–7 ^a^*	0–10	0–5 ^a^*	0.041
Phenotype n (%)					χ^2^ = 136.6; <0.001
Allergic	1 (2)	2 (4.1)	44 (89.8)	2 (4.1)	
Eosinophilic	72 (67.3)	20 (18.7)	3 (2.8)	12 (11.2)	
Combined	32 (39.5)	23 (28.4)	19 (23.5)	7 (8.6)	
Admission to hospital n (%)					χ^2^ = 2.4; 0.486
No	38 (86.4)	15 (78.9)	27 (84.4)	10 (100)	
Yes	6 (13.6)	4 (21.1)	5 (15.6)	0 (0)	
Headache n (%)					χ^2^ = 9.4; 0.024
No	21(47.7)	15 (78.9)	19 (59.4)	9 (90.0)	
Yes	23 (52.3)	4 (21.1)	13 (40.6)	1 (10.0)	
Fever n (%)					χ^2^ = 8.3; 0.040
No	5 (11.4)	7 (36.8)	11 (34.4)	4 (40.0)	
Yes	39 (88.6)	12 (63.2)	21 (65.6)	6 (60.0)	

^a, aa, aaa^ *p* < 0.05, 0.01, 0.001, respectively, vs. benralizumab; ^b^ *p* < 0.05 vs. mepolizumab; ^a^*—borderline significance vs. benralizumab; *p* according to ANOVA or Kruskal–Wallis ANOVA followed by Mann–Whitney U test; χ^2^ test.

**Table 3:** Disease severity and treatment options (ambulatory, hospital) and basic clinical characteristics.

Parameter	Ambulatory Treated n = 90	Hospital Admission n = 15	*p*
Age, years	53.30 ± 12.5	56.20 ± 13.7	Ns
Biological therapy duration (months)	22.8 ± 16.1	16.6 ± 15.0	Ns
Exacerbation number before COVID-19	0–10	0–5	Ns
FEV1%	74.2 ± 21.6	59.7 ± 13.2	0.014
Oxygenation therapy, days	/	5 (0–13)	/
Therapy			χ^2^ = 25.3 *p* < 0.001
without therapy	73 (81.1)	8 (53.3)	
O_2_	0 (0)	1 (6.7)	
CS	17 (18.9)	3 (20.0)	
combination O_2_ + CS	0 (0)	3 (20.0)	
Days SCS during COVID-19	/	13.0 (2.5–22.5)	/
LMWH			χ^2^ = 59.9 *p* < 0.001
NO	89 (98.9)	5 (33.3)	
YES	1 (1.1)	10 (66.7)	
Hospitalization, days	/	11.5 (7.0–16.0)	/
Non-invasive ventilation, days	/	0	/
Mechanical ventilation, days	/	1 pat. 7 days; 1 pat. 14 days	/

Mann–Whitney U test for continuous variables, χ^2^ test for categorical variables Mann–Whitney U test for continuous variables, χ^2^ test for categorical variables. CS- corticosteroids and SCS—systemic corticosteroids.

**Table 4:** COVID-19 quarters’ period comparison according to vaccination status, COVID-19-related hospitalization, and implemented therapy.

Variable	First (March 2020–September 2020)	Second (October 2020–March 2021)	Third (April 2021–October 2021)	Fourth (November 2021–April 2022)	χ^2^, *p*
Vaccinal status, n (%)					2.4, 0.499
No	2 (50)	8 (25)	2 (14.3)	12 (21.8)	
Yes	2 (50)	24 (75)	12 (85.7)	43 (53.1)	
Hospitalization, n (%)					5.5, 0.139
No	2 (50)	29 (90.6)	11 (78.6)	48 (87.3)	
Yes	2 (50)	3 (9.4)	3 (21.4)	7 (12.7)	
Oxygen therapy during COVID-19, n (%)					3.3, 0.343
No	3 (75)	29 (90.6)	11 (78.6)	51 (92.7)	
Yes	1 (25)	3 (9.4)	3 (21.4)	4 (7.3)	
Systemic corticosteroid therapy during COVID-19, n (%)					4.1, 0.250
No	1 (25)	20 (62.5)	8 (57.1)	39 (70.9)	
Yes	3 (75)	12 (37.5)	6 (42.9)	16 (29.1)	
Oxygen and systemic corticosteroids during COVID-19, n (%)					1.5, 0.683
No	2 (66.7)	9 (75)	3 (50)	12 (75)	
Yes	1 (33.3)	3 (25)	3 (50)	4 (25)	

χ^2^ test for categorical variables.

**Table 5:** Comparison of the before vaccination–after vaccination status of COVID-19 disease among the period quarters and between the vaccinal statuses (first, second, and third (booster) vaccine).

Quarter Period of COVID-19 Disease	I Vaccine Before/After/Nonvaccinated + Unknown	II Vaccine Before/After/Nonvaccinated + Unknown	III Vaccine Before/After/Nonvaccinated + Unknown	χ^2^, *p*
I (n = 4)	4/0/0	2/0/2	1/0/3	0.635, 0.721
II (n = 33)	14/1/8	0/20/10 + 3	0/13/20	0.101, 0.950
III (n = 13)	3/6/1 + 3	8/2/1 + 3	0/2/10 + 1	9.4, <0.01
IV (n = 55)	5/32/12 + 6	1/37/13 + 4	2/8/42 + 3	34.9, <0.001
χ^2^, *p*	32.2, <0.001	54.9, <0.001	17.8, <0.001	/

χ^2^ test for categorical variables; quarter periods: I to September 2020; II October 2020–March 2021; III April 2021–October 2021; IV November 2021–April 2022.

## Data Availability

The original contributions presented in this study are included in the article. Further inquiries can be directed to the corresponding author(s).
